# Longitudinal Patterns of Brain Parenchymal Damage Associated With the ALPS Index in a Community‐Based Cohort

**DOI:** 10.1002/cns.71107

**Published:** 2026-08-25

**Authors:** Chengqian Li, Wen‐Xin Li, Zi‐Yue Liu, Fei‐Fei Zhai, Fei Han, Ming‐Li Li, Li‐Xin Zhou, Jun Ni, Ming Yao, Shu‐Yang Zhang, Li‐Ying Cui, Zheng‐Yu Jin, Yi‐Cheng Zhu

**Affiliations:** ^1^ Department of Neurology, State Key Laboratory of Complex Severe and Rare Diseases Peking Union Medical College Hospital, Chinese Academy of Medical Sciences and Peking Union Medical College Beijing China; ^2^ Department of Radiology, State Key Laboratory of Complex Severe and Rare Diseases Peking Union Medical College Hospital, Chinese Academy of Medical Sciences and Peking Union Medical College Beijing China; ^3^ Department of Cardiology, State Key Laboratory of Complex Severe and Rare Diseases Peking Union Medical College Hospital, Chinese Academy of Medical Sciences and Peking Union Medical College Beijing China

**Keywords:** brain atrophy, diffusion tensor imaging along the perivascular space, glymphatic dysfunction, longitudinal study, white matter microstructure

## Abstract

**Aims:**

To better comprehend relationships between the analysis along the perivascular space (ALPS) index and glymphatic dysfunction, we assessed the spatiotemporal patterns of structural brain injury related to the ALPS index in a longitudinal study.

**Methods:**

We performed voxel/vertex‐wise analysis to explore the relationship between lower ALPS index and brain parenchymal injury. We also performed brain network connectivity analysis to investigate the relationship between white matter disruption and cortical atrophy.

**Results:**

The lower baseline ALPS index showed an association with drastic progression of disruption of white matter microstructural integrity, primarily located in the subcortical regions where association fibers are distributed. Brain network connectivity analysis revealed an association of the lower ALPS index with a disconnected sub‐network in the midline and right frontal lobe, where association fibers are widely distributed. The lower ALPS index also displayed a correlation with cortical atrophy in the projection areas of both association and projection fibers in cross‐sectional analysis. However, this correlation was nonsignificant in longitudinal analysis.

**Conclusion:**

The ALPS index could be considered a composite marker of perivascular dynamics and white matter microstructural integrity, shedding light on the spatial pattern of disruption of white matter integrity in community‐dwelling populations.

## Graphical abstract

In our community‐based cohort, a lower baseline ALPS index is primarily associated with the progression of white matter microstructural disruption. Cortical atrophy occurs mainly in projection areas of affected white matter fibers. Therefore, the ALPS index represents a composite marker of perivascular dynamics and white matter microstructural integrity, rather than a direct indicator of glymphatic function.

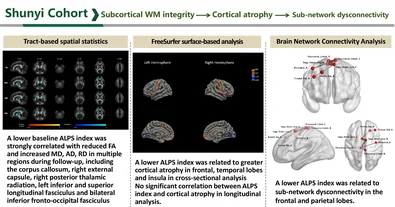

## 1 Introduction

The glymphatic system, a central nervous system‐specific network discovered recently, enables cerebrospinal fluid (CSF) exchange with interstitial fluid [1, 2]. Currently, this system has become the hot topic of research on brain clearance mechanisms [3, 4, 5]. By conducting an in vivo study in a rodent model through two‐photon imaging, Iliff et al. in 2012 reported the entry of the CSF into the parenchyma from periarterial spaces and clearance of interstitial fluid through perivenous routes [6]. However, the glymphatic system may show disparities between humans and rodent models; moreover, invasive imaging techniques are impractical for routine use in population‐level exploration.

Taoka et al. in 2017 introduced the diffusion tensor imaging (DTI) along the perivascular space (ALPS) index for evaluating glymphatic system function [7]. In paraventricular regions, the medullary perivascular spaces run aligned in *x*‐axis direction, perpendicular to association fibers (*y*‐axis direction) and projection fibers (*z*‐axis direction). The ALPS index is computed by calculating the ratio of *x*‐axis diffusivity, which runs parallel to presumed glymphatic flow, to *y*‐ and *z*‐axis diffusivities of association and projection fibers. Although this index is technically simple, suitable for prospective and longitudinal analyses, and compatible with standard magnetic resonance imaging (MRI) protocols, its application remains controversial as it might be influenced not only by glymphatic flow but also by the microstructural asymmetry of white matter tensor [8, 9, 10].

Therefore, we hypothesized that the ALPS index might be a composite imaging marker influenced by both glymphatic function and white matter microstructural integrity. However, the spatiotemporal patterns of cortical atrophy and white matter damage related to the ALPS index remain insufficiently investigated in aging populations, and further attention should be paid to the potential role of neurodegenerative processes. To comprehensively elucidate the relationship between glymphatic dysfunction and the ALPS index, we assessed the associations of the ALPS index with cortical atrophy and white matter microstructural disruption in a longitudinal community‐based cohort.

## 2 Methods

### 2.1 Participant Details and Study Design

This investigation is a component of the Shunyi cohort study, which is a current, community‐based, prospective cohort study for determining risk factors as well as related brain imaging of aging‐related, cerebrovascular and cardiovascular diseases. Both study design and methods utilized in the Shunyi study are reported earlier [11]. Between June 2013 and April 2016, a total of 1586 participants underwent baseline assessments, including structured questionnaires, physical examinations, and blood tests. Among them, 362 participants who did not complete baseline brain MRI scans and 79 participants whose MRI data quality was insufficient for ALPS index calculation or brain structural segmentation were excluded. We further excluded participants with a history of tumors (*n* = 12). Because stroke lesions may affect the accuracy of ALPS index calculation and brain structural segmentation, participants with a history of stroke were also excluded (*n* = 13). Finally, 1120 participants were included in the cross‐sectional analysis.

All participants included in the cross‐sectional analysis were invited to undergo a 5‐year follow‐up brain MRI. Follow‐up brain MRI scans were performed between September 2019 and October 2021 at the same imaging center as the baseline MRI scans. Among the 1120 participants included in the cross‐sectional analysis, 422 participants did not complete follow‐up brain MRI and were therefore excluded from the longitudinal analysis. In addition, 16 participants who had incident stroke during the follow‐up period were excluded. Finally, 682 participants were included in the longitudinal analysis. The study flow diagram is shown in Figure S1.

### 2.2 MRI Protocol

Baseline MRI was conducted from July 2014 to April 2016, while follow‐up MRI was conducted between September 2019 and October 2021. Baseline as well as follow‐up MRI data were obtained using the same 3 T Skyra scanner (Siemens, Erlangen, Germany) and protocols based on previously reported parameters [11]. Three‐dimensional T1‐weighted imaging, T2‐weighted imaging, DTI, and fluid‐attenuated inversion recovery imaging (FLAIR) were conducted as mentioned in Supporting Information. All imaging data were preprocessed with the processing pipeline from the UK Biobank, based on our MRI parameters [12].

### 2.3 Automated ALPS Index Calculation

To calculate ALPS, an automated method was developed and validated against manual measurements in our previous investigations [13]. Four spherical regions of interest (sROIs, 3 mm radius) were automatically positioned on bilateral association and projection fibers in the Montreal Neurological Institute (MNI) 152 space. For these sROIs, the center coordinates were (37, −12, 24), (26, −12, 24), (−26, −12, 24), and (−37, −12, 24). Association fiber masks (corticofugal corona radiata tract fibers) and projection fiber masks (superior longitudinal fasciculus) were chosen in accordance with the Johns Hopkins University‐International Consortium for Brain Mapping‐labels‐1 mm template in the MNI152 space. The DTI fitting tool (an FSL component) was used to generate fractional anisotropy (FA) images from raw DTI data [14]. Projections, association fiber masks, and sROI masks were then transformed into FA images for each participant. For the sROIs, the maximum range values of *y*‐ and *z*‐axis coordinates were separately obtained for each hemisphere, relative to the transformed sROI and FA images. The intersection of the sROI cuboid and transformed fiber masks against the FA image was subsequently selected as the ROIs to automatically calculate ALPS in each participant's space. Finally, a previously established method was implemented to derive the ALPS index [7]. The mean diffusivities along the x‐axis of association and projection fibers, *y*‐axis of projection fibers, and *z*‐axis of association fibers in the ROIs were recorded as Dxassoc, Dxproj, Dyproj, and Dzassoc, respectively. The left and right ALPS indices for each side were determined as follows: [(Dxproj+Dxassoc)/(Dyproj+Dzassoc)]. For both hemispheres, the ALPS indices were separately estimated and averaged.

### 2.4 DTI Data Processing

FSL v6.0.7 (FMRIB Software Library, https://fsl.fmrib.ox.ac.uk/fsl/docs/#/) was utilized for processing DTI images in baseline and follow‐up analyses in the same server environment. The DTI data were preprocessed according to the UK Biobank diffusion processing pipeline, which included skull stripping, correction of eddy current, head motion and gradient distortion, and spatial alignment normalization [12]. All voxels in the generated white matter masks, with a FA threshold of > 0.2, were used for determining global mean DTI metrics. Subsequently, axial diffusivity (AD), mean diffusivity (MD), radial diffusivity (RD), and global mean FA, which indicate white matter microstructural integrity, were calculated for each participant.

### 2.5 Assessment of White Matter Hypersensitivity (WMH)

Baseline and follow‐up WMH volumes were segmented and derived using the Brain Intensity Abnormality Classification Algorithm (BIANCA), which is a tool for automated segmentation to classify voxels according to spatial features and relative intensity, in the same server environment [15]. For 25 participants, we manually labeled WMH on the FLAIR sequences to train the segmentation algorithm using BIANCA. This algorithm's output is an individual probability map threshold at 0.95 for generating a binary WMH map for extracting WMH volume (mL). Compared to manual segmentation, the interclass correlation coefficient and the mean dice coefficient in our dataset were 0.94 and 0.87, respectively.

### 2.6 Assessment of Atrophy Measures

To determine brain parenchymal atrophy, Freesurfer v8.1 (https://surfer.nmr.mgh.harvard.edu/) was utilized for developing an automatic segmentation pipeline in the native space for extracting brain parenchymal volumes (white matter, gray matter, and hippocampus). The total intracranial volume (sum of volumes of white matter, gray matter, and CSF) was segmented automatically with Statistical Parametric Mapping 12 (https://www.fil.ion.ucl.ac.uk/spm/). For calculating brain parenchymal fraction (BPF), the sum of volumes of gray matter and white matter was divided by total intracranial volume. White matter fraction (WMF), hippocampus fraction, and gray matter fraction (GMF) were obtained as the white matter volume, bilateral hippocampal volume, and gray matter volume divided by total intracranial volume, respectively. To comprehensively describe cortical morphology for vertex‐wise analysis, we also extracted cortical thickness and cortical volume using Freesurfer v8.1. The section “Statistical image analysis” includes a detailed description of vertex‐wise analysis method.

### 2.7 Assessment of Topological Properties

GraphVar 2.03a (https://www.nitrc.org/projects/graphvar/), a tool included in MATLAB R2020b (https://mathworks.com), was utilized for graph analyses of the brain white matter connectivity network [16, 17]. The details of network calculations and network‐based statistics have been described in our previous publications [18]. For all participants, we constructed a weighted edge through multiplication of reconstructed fiber number and mean FA along the fiber bundle connecting the two regions.

To investigate the cortical and subcortical regions of network disconnection in relation to the lower ALPS index, we carried out generalized linear model (GLM) analysis using the ALPS index and each edge's strength. A total of 5000 permutation tests were conducted, with the significance threshold considered at Bonferroni‐corrected *p*‐value (< 0.05). We then used BrainNet Viewer (https://www.nitrc.org/projects/bnv/) to graphically visualize the sub‐network containing the determined significant connections [19]. The significant nodes and edges derived from the statistical analysis were superimposed on a standard anatomical brain surface mesh provided with the BrainNet Viewer.

### 2.8 Covariate Assessment

Clinical as well as demographic data, such as sex, age, hyperlipidemia, hypertension, diabetes mellitus (DM), and alcohol consumption and smoking history, were obtained from physical examination and structured questionnaires. These covariates are defined in Supporting Information.

### 2.9 Statistical Global Data Analysis

Demographic features, baseline neuroimaging features, and their annual variations were subjected to descriptive analyses during the follow‐up period for the cross‐sectional and longitudinal analyzed populations. Continuous variables were represented by mean and standard deviation or median and interquartile range (IQR). Frequencies and proportions were utilized for expressing categorical variables. Baseline characteristic differences between participants who completed the follow‐up protocol and those who could not complete this protocol were analyzed by *t*‐test and chi‐square test for continuous and categorical variables, respectively.

In cross‐sectional analysis, relationships of brain structural measurements and DTI metrics with the ALPS index were evaluated with multiple linear regression models by using the ALPS index as the independent variable and DTI parameters or brain measures as the dependent variables. Covariates were adjusted sequentially using three models. Model 1 was univariate. For Model 2, sex and age were adjusted. For Model 3, the following parameters were adjusted: sex, age, and vascular risk factors (hyperlipidemia, DM, hypertension, and alcohol consumption and smoking history).

In longitudinal analysis, baseline and follow‐up neuroimaging measurements were jointly modeled as repeated‐measures outcomes. Generalized linear mixed models were used to examine whether the baseline ALPS index was associated with subsequent changes in neuroimaging measures. Repeated measurements from the same participant were accounted for by including participant‐specific random intercepts, with time point modeled as a within‐participant repeated factor. Time was coded as baseline and follow‐up, and the interaction term between baseline ALPS index and time was used to assess whether baseline ALPS predicted longitudinal changes in DTI parameters or brain structural measurements.

Three models were constructed with sequential adjustment for covariates, as in the cross‐sectional analysis. Model 1 was unadjusted. Model 2 was adjusted for baseline age and sex. Model 3 was further adjusted for baseline vascular risk factors, including hypertension, DM, hyperlipidemia, smoking history, and alcohol consumption.

Given the positively skewed distribution of white matter microstructural measurements (a common characteristic of DTI metrics), Gamma generalized linear mixed models (Gamma‐GLMMs) with a log link function were employed to determine the associations between the baseline ALPS index and DTI parameters (FA, AD, RD, and MD). To interpret effects in the original metric of DTI parameters, we computed relative ratios (RRs) as well as their corresponding 95% confidence intervals (CIs) by exponentiating the log‐scale coefficients (and their 95% CIs). The statistical significance of fixed effects was determined by Wald tests (Type III).

Brain structural measurements (continuous proportional outcome variables including BPF, GMF, WMF, hippocampus fraction, and WMH fraction, each constrained to the range [0, 1]) were analyzed using beta generalized linear mixed models (Beta GLMMs) with a logit link function. To interpret effects in the original metric of proportional outcomes, absolute changes in proportion (Abs. Change) and their corresponding 95% CIs were calculated by back‐transforming the logit‐scale coefficients and their 95% CIs using the inverse logit function. For non‐intercept terms, the absolute change in proportion was further derived by subtracting the reference‐level proportion from the back‐transformed proportional effect. The statistical significance of fixed effects was assessed by Wald tests (Type III).

R software (v4.3.3) was utilized for performing statistical analyses. Correction for multiple testing in all three models was achieved using false discovery rate (FDR) with the Benjamini‐Hochberg approach. For each model, FDR correction was applied across multiple neuroimaging outcomes, and an FDR‐corrected *p*‐value < 0.05 was considered statistically significant. For the abovementioned models, we confirmed the assumptions based on residual diagnosis plots.

### 2.10 Statistical Image Analysis

Voxel‐wise analysis and vertex‐wise analysis were implemented for determining correlations of brain parenchymal damage with the ALPS index.

Tract‐Based Spatial Statistics (TBSS, https://fsl.fmrib.ox.ac.uk/fsl/docs/#/diffusion/tbss) implemented in FSL was utilized in voxel‐wise analysis of DTI data [20]. For cross‐sectional analysis, baseline DTI data were preprocessed following the standard TBSS pipeline. For longitudinal analysis, both baseline and 5‐year follow‐up DTI data were included. Follow‐up FA, AD, RD, and MD maps were rigidly registered to their corresponding baseline FA maps using flirt with 6 degrees of freedom to represent inter‐scan motion. The registered follow‐up metrics were subsequently projected onto the baseline‐derived mean FA skeleton through tbss_non_FA. Annualized change maps for each metric were computed by subtracting the baseline skeletonized values from the follow‐up values and dividing by the 5‐year interval. A statistical inference tool (‘randomize’) involving permutation for a nonparametric approach was employed for GLM analysis of cross‐sectional (baseline metrics) and longitudinal (annualized change) data. A total of 5000 permutation tests were conducted, and threshold‐free cluster enhancement (TCFE) corrected *p* < 0.05 was considered the significance threshold, with adjustments made for sex and age.

The relationship of cortical volume and thickness with the ALPS index was evaluated by surface‐based analysis using FreeSurfer v8.1. The standard recon‐all pipeline was implemented for processing structural MRI data in cross‐sectional analysis. Paired baseline and follow‐up MRI scans were evaluated with FreeSurfer longitudinal pipeline in longitudinal analysis. First, a subject‐specific template was developed for each participant using recon‐all with the –base option, followed by longitudinal reconstruction (–long) for both time points to minimize scan‐related bias. Annual percentage change in cortical thickness and volume was computed for each vertex. GLM was used to elucidate how cortical atrophy rates associated with the baseline ALPS index were spatially distributed. Cluster‐wise significance was determined by Monte Carlo simulation (number of permutations = 5000) at a cluster‐forming threshold of 2.0 and a cluster‐wise *p*‐value (CWP) of < 0.05, with adjustment for total intracranial volume.

## 3 Results

### 3.1 Demographics

A total of 1120 participants with complete baseline data were included in the cross‐sectional analysis. The mean age of these participants was 56.99 ± 9.26 years, and 35.18% were men. Among them, 682 participants (60.9%) completed follow‐up brain MRI and were included in the longitudinal analysis. The mean follow‐up interval was 5.52 ± 0.48 years. At baseline, participants included in the longitudinal analysis had a mean age of 55.02 ± 7.98 years, and 32.70% were men. The baseline characteristics and longitudinal changes of the study population are summarized in Table 1.

**TABLE 1:** Demographic characteristics of the longitudinal analyzed population (*n* = 682) at baseline and follow‐up.

Variables	Total (*n* = 682)
Demographic characteristics	
Age, mean (SD), year	55.02 (7.98)
Male, No. (%)	223 (32.70)
Hypertension, No. (%)	326 (47.80)
Diabetes mellitus, No. (%)	86 (12.61)
Hyperlipidemia, No. (%)	318 (46.63)
Smoking history, No. (%)	124 (18.18)
Alcohol consumption history, No. (%)	148 (21.70)
Follow‐up years, mean (SD)	5.52 (0.48)
Baseline neuroimaging characteristics	
ALPS index	1.42 (0.12)
BPF, mean (SD), %	75.69 (2.59)
GMF, mean (SD), %	34.60 (1.53)
WMF, mean (SD), %	32.36 (1.77)
Hippocampus fraction, mean (SD), %	0.57 (0.05)
WMH volume, median (interquartile range), mL	7.89 (7.50, 8.38)
Fractional anisotropy, mean (SD)	0.40 (0.01)
Mean diffusivity, mean (SD), ×10^−3^ mm^2^/s	0.79 (0.02)
Axial diffusivity, mean (SD), ×10^−3^ mm^2^/s	1.16 (0.02)
Radial diffusivity, mean (SD), ×10^−3^ mm^2^/s	0.61 (0.02)
Annual change during follow‐up interval	
BPF, mean (SD), %	−0.28 (0.39)
GMF, mean (SD), %	−0.17 (0.21)
WMF, mean (SD), %	−0.11 (0.20)
Hippocampus fraction, mean (SD), ×10^−1^, %	−0.03 (0.03)
WMH volume, median (interquartile range), mL	0.08 (0.05, 0.12)
Fractional anisotropy, mean (SD), ×10^−1^	−0.002 (0.009)
Mean diffusivity, mean (SD), ×10^−4^ mm^2^/s	0.007 (0.032)
Axial diffusivity, mean (SD), ×10^−4^ mm^2^/s	0.010 (0.040)
Radial diffusivity, mean (SD), ×10^−4^ mm^2^/s	0.006 (0.029)

*Note:* The annual change for continuous variables was calculated as (follow‐up value—baseline value)/follow‐up years.

Abbreviations: ALPS, diffusion along the perivascular space; BPF, brain parenchymal fraction, the sum of gray matter and white matter volumes/total intracranial volume; GMF, gray matter fraction, gray matter volume/total intracranial volume; Hippocampus fraction, hippocampus volume/total intracranial volume; SD, standard deviation; WMF, white matter fraction, white matter volume/total intracranial volume; WMH, white matter hyperintensity.

The mean baseline ALPS index was 1.41 ± 0.13 in the overall cross‐sectional analysis sample and 1.42 ± 0.12 in the longitudinal analysis sample, with no significant difference between the two samples. Among the 682 participants included in the longitudinal analysis, the mean baseline BPF was 75.69% ± 2.59%, with a mean annual decrease of 0.28% ± 0.39% during follow‐up. The mean baseline FA was 0.40 ± 0.01, with a mean annual decrease of 0.0002 ± 0.0009. The baseline median WMH volume was 7.89 mL (IQR: 7.50–8.38 mL). During follow‐up, WMH volume showed a median annual increase of 0.08 mL (IQR: 0.05–0.12 mL).

Compared to patients with follow‐up MRI, those without follow‐up MRI were aged, inclined to be males, had a greater prevalence of exposure to risk factors for cardiovascular diseases, had a larger burden of WMH volume, and had high severity of brain atrophy (Table S1).

### 3.2 Correlation of White Matter Damage and the ALPS Index

The baseline and longitudinal associations of WMH volume and global mean DTI metrics with the ALPS index are shown in Tables S2 and S3. In cross‐sectional analysis, the low ALPS index exhibited a stronger association with global mean DTI metrics and WMH volume. In longitudinal analysis, following adjustments for sex, vascular risk factors (DM, smoking and alcohol consumption history, hypertension, and hyperlipidemia), and age, those with lower baseline ALPS index had a greater decrease in FA (RR = 1.020, 95% CI = 1.007–1.033, *p* = 0.003) and considerable increase in MD (RR = 0.972, 95% CI = 0.956–0.988, *p* < 0.001), RD (RR = 0.967, 95% CI = 0.947–0.987, *p* = 0.001), AD (RR = 0.978, 95% CI = 0.965–0.991, *p* < 0.001), and WMH fraction (Abs. Change = −0.040, 95% CI = −0.055, −0.025, *p* < 0.001) in a linear mixed effects model, indicating diffused white matter microstructural integrity disruption. Importantly, the ALPS index significantly positively interacted with time (Abs. Change = 0.009, 95% CI = 0.001–0.016, *p* = 0.037), indicating that the ALPS index's protective effect against WMH volume was attenuated over the 5‐year duration of follow‐up.

**FIGURE 1:**
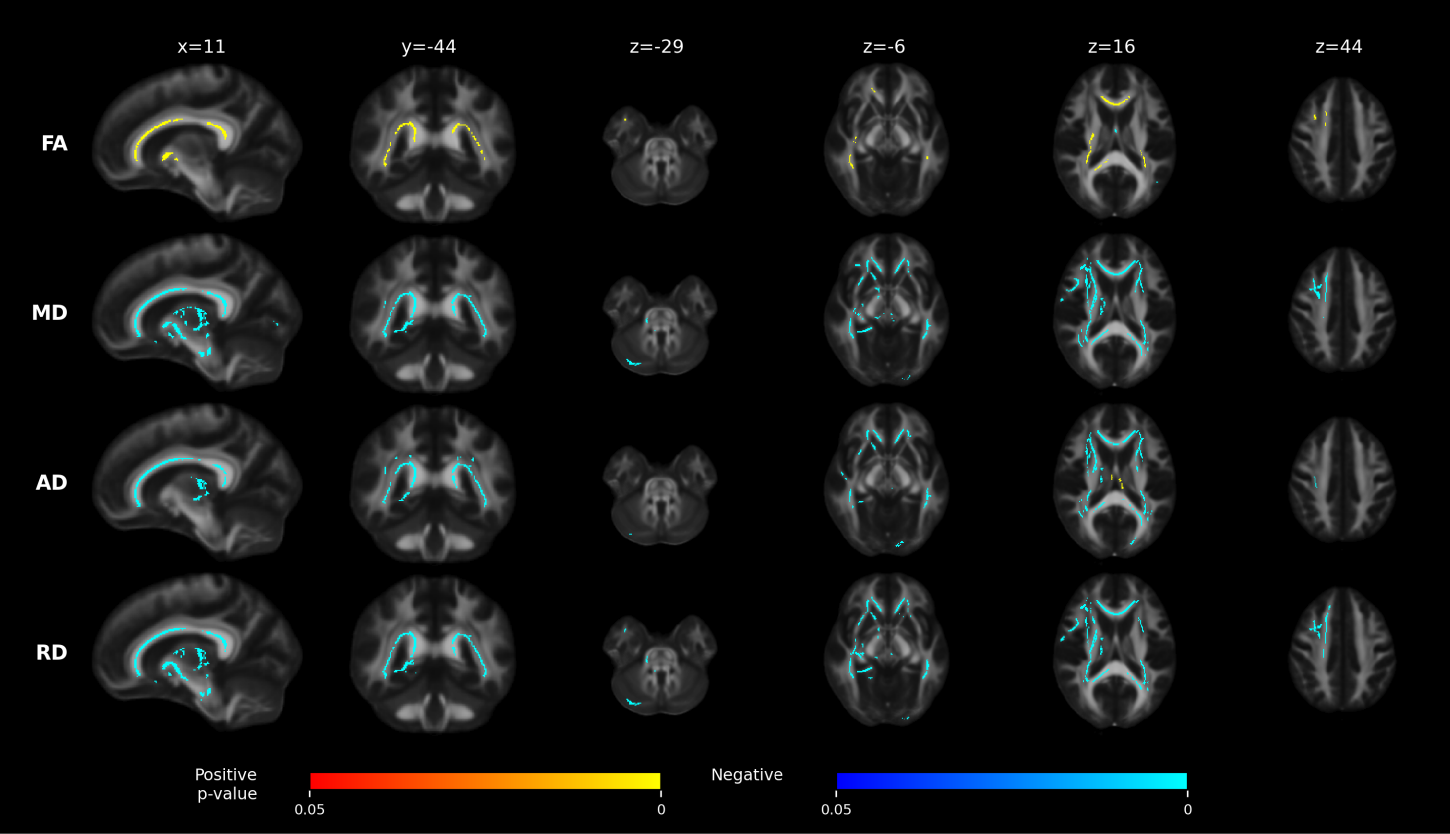
Distribution of progression of white matter microstructural damage correlated to baseline ALPS index. The red‐yellow map and blue map show locations on the white matter skeleton where faster FA decrease, or faster increase in MD, AD, and RD is correlated with lower baseline ALPS index, respectively (threshold‐free cluster enhancement corrected *p* < 0.05, adjusted for age and sex). AD axial diffusivity; ALPS, diffusion along the perivascular space; FA, fractional anisotropy; MD, mean diffusivity; RD, radial diffusivity.

The TBSS analysis of the baseline and longitudinal associations between DTI metrics and the ALPS index is presented in Figure S2 and Figure 1. Table S4 and Table 2 show voxel clusters with significant correlations. In cross‐sectional analysis, a low ALPS index was strongly correlated with higher MD, AD, and RD and a lower FA in several regions on the white matter skeleton (Figure S2). In longitudinal analysis (Figure 1), as shown by TBSS analysis, the spatial pattern of disruption of white matter microstructural integrity exhibited a relationship with the lower baseline ALPS index. We found that the lower baseline ALPS index correlated greatly with increased MD, AD, and RD and decreased FA in several regions during follow‐up, including the right external capsule, corpus callosum, a portion of the left inferior and superior longitudinal fasciculus and bilateral inferior fronto‐occipital fasciculus, and right posterior thalamic radiation (TFCE corrected *p* < 0.05, with adjustment for sex and age, Table 2).

**TABLE 2:** Listing of progression of white matter fiber tracts damage correlated to lower baseline ALPS index.

DTI parameters	White matter fiber tracts (JHU WM tractography atlas)	Voxel coordinates of local maxima (MNI coordinates)			Cluster size (Voxels)	*t* score	*p*
		X	Y	Z			
FA	Splenium of corpus callosum	−17.0	−37.0	28.0	3932	6.722	< 0.05
	Genu of corpus callosum	9.0	14.0	23.0	1516	4.543	< 0.05
	Sagittal stratum L (include inferior longitidinal fasciculus and inferior fronto‐occipital fasciculus)	−39.0	−39.0	−11.0	93	4.231	< 0.05
	External capsule R	35.0	−8.0	2.0	91	4.797	< 0.05
	Inferior fronto‐occipital fasciculus R	36.0	−16.0	−8.0	60	4.716	< 0.05
	Body of corpus callosum	−7.0	8.0	25.0	53	3.529	< 0.05
MD	Body of corpus callosum	7.0	−33.0	22.0	25,626	1.557	< 0.05
AD	Body of corpus callosum	37.0	−46.0	−6.0	17,663	1.625	< 0.05
	Posterior thalamic radiation R (include optic radiation)	31.0	−59.0	0.0	153	2.822	< 0.05
	Superior longitudinal fasciculus L	−37.0	−54.0	17.0	101	2.926	< 0.05
RD	Body of corpus callosum	27.0	−11.0	32.0	22,739	1.630	< 0.05

*Note:* Cluster sizes and locations for voxels with significantly decreased FA and increased MD, AD, RD values correlated to lower baseline ALPS index. *p*‐value was adjusted for age and sex. The cluster with low size (voxels < 50) has been excluded.

Abbreviations: AD, axial diffusivity; ALPS, diffusion along the perivascular space; DTI, diffusion tensor imaging; FA, fractional anisotropy; JHU WM tractography atlas, John Hopkins University white‐matter tractography atlas; L, left hemisphere; MD, mean diffusivity; MNI, Montreal Neurological Institute; R, the right hemisphere; RD, radial diffusivity.

### 3.3 Correlation of the ALPS Index With Brain Atrophy

The baseline and longitudinal associations between brain structural measurements (BPF, GMF, WMF, and hippocampus fraction) and the ALPS index are summarized in Table S3. A strong association was noted between the lower ALPS index and BPF, GMF, WMF, and hippocampus fraction in cross‐sectional analysis, with the same results in all the models. In longitudinal analysis, following adjustments for sex, age, and previously mentioned vascular risk factors, significant associations were noted between the baseline ALPS index and the progression of BPF (Abs. Change = 0.037, 95% CI = 0.008–0.066, *p* = 0.015), WMF (Abs. Change = 0.026, 95% CI = 0.007–0.045, *p* = 0.012), and hippocampus fraction (Abs. Change = 0.022, 95% CI = 0.009–0.036, *p* = 0.003) during the follow‐up interval. Notably, the ALPS index significantly positively interacted with time for both WMF (Abs. Change = 0.010, 95% CI = 0.002–0.019, *p* = 0.037) and hippocampus fraction (Abs. Change = 0.006, 95% CI = 0.001–0.011, *p* = 0.037). These observations indicate that higher ALPS levels are linked to greater long‐term preservation of both white matter and hippocampus volumes. However, the ALPS index did not exhibit any remarkable association with GMF progression.

**FIGURE 2:**
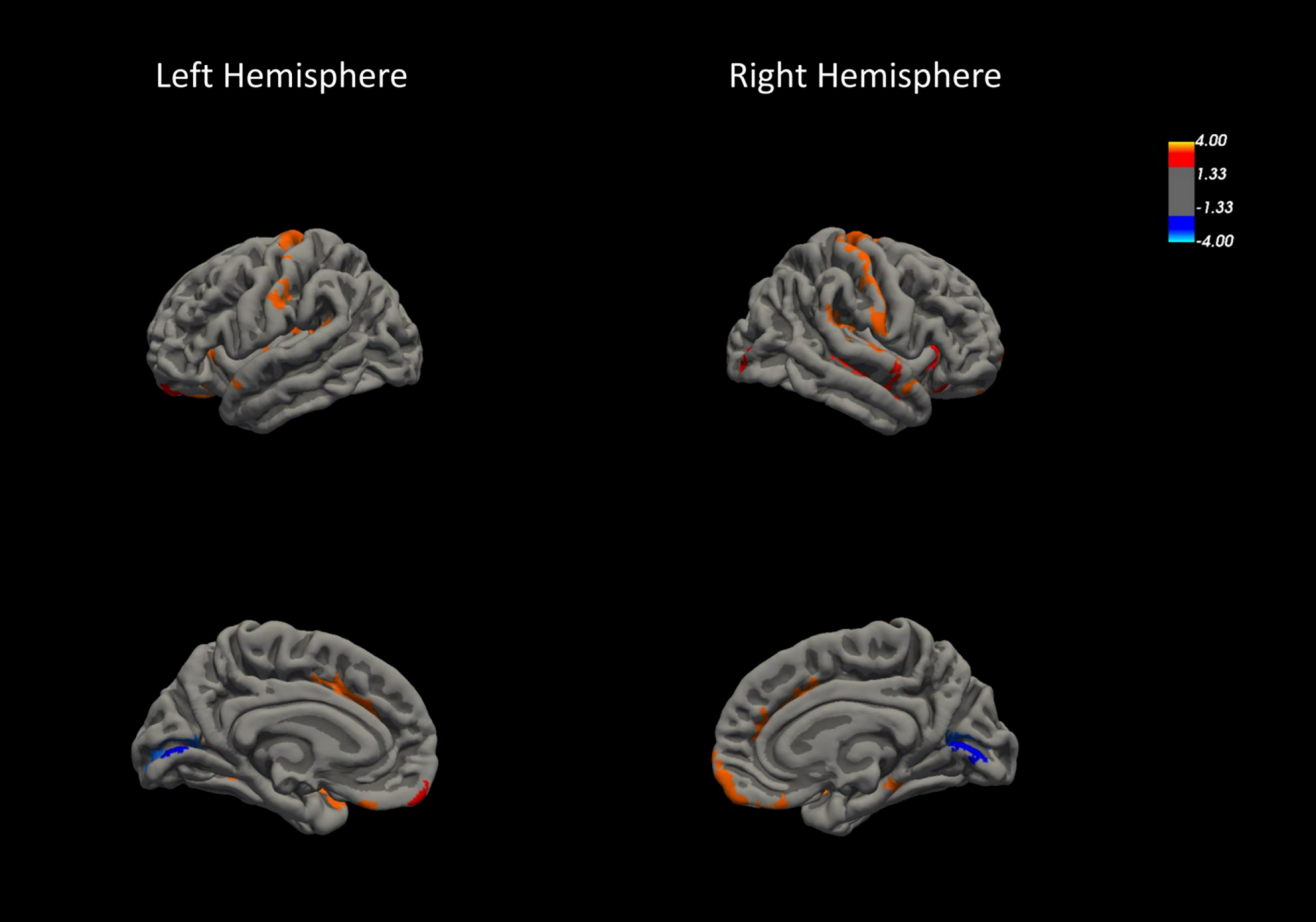
Distribution of cortical thickness regions with significant correlation to ALPS index. Clusters of cortical thickness significantly associated with ALPS index, including bilateral superior frontal, bilateral precentral, right medial orbitofrontal, left superior temporal, and right insula (Bonferroni‐corrected with Monte Carlo simulation for cluster *p*‐value < 0.05 and adjusted for total intracranial volume). The displayed color bar is in the logarithmic scale of *p*‐values (−log_10_ [*p*‐value]). ALPS, diffusion along the perivascular space.

Figures 2 and S3 display brain atrophy spatial distribution in relation to the ALPS index. Evaluation of the baseline brain volume by vertex‐wise analysis revealed a remarkable association of the lower ALPS index with cortical thickness shrinkage in the bilateral superior frontal, bilateral precentral, right medial orbitofrontal, left superior temporal, and right insula (Bonferroni‐corrected with Monte Carlo simulation for cluster *p*‐value < 0.05, with adjustment for total intracranial volume, Figure 2). The lower ALPS index also displayed a prominent correlation with a smaller cortical volume of clusters in the right postcentral, right medial orbitofrontal, bilateral superior temporal, and right transverse temporal (Figure S3). Details of the critical clusters are given in Table 3, including MNI coordinates, statistics, and cluster size. However, in longitudinal analysis, no significant clusters for the regions of progression of brain atrophy related to the lower baseline ALPS index were identified (Table S5).

**TABLE 3:** Distribution of brain atrophy with significant correlation to lower ALPS index.

Brain regions	MNI coordinates			Size (mm^2^)	Effect size	*p*
	X	Y	Z			
Cortical thickness						
Frontal lobe						
Right precentral	34.4	−17.4	37.3	650.89	8.289	0.0004
Right precentral	8.7	−29	68.5	529.65	8.855	0.0004
Left superiorfrontal	−11.8	11.9	39.8	481.58	8.026	0.0004
Right medialorbitofrontal	9.9	33.1	−20.1	478.52	13.364	0.0004
Right superiorfrontal	12.9	21.3	35.6	447.09	7.062	0.0004
Left precentral	−12.5	−27.5	67.4	424.69	10.533	0.0004
Left lateralorbitofrontal	−29	32.3	−10.5	268.30	8.305	0.0004
Right lateralorbitofrontal	33.8	34.2	−7.5	246.51	7.440	0.0008
Left medialorbitofrontal	−7	37.5	−22.8	242.96	9.886	0.0004
Parietal lobe						
Left postcentral	−60.7	−14.5	28.2	226.32	8.713	0.0004
Occipital lobe						
Right pericalcarine	8.9	−67.6	12.1	342.19	−10.612	0.0004
Left pericalcarine	−12.4	−69.5	8.5	253.09	−10.768	0.0004
Right lateraloccipital	36.1	−87.8	−4.7	200.07	4.932	0.002
Temporal lobe						
Left superiortemporal	−47.9	−8.5	−12.5	2237.83	17.604	0.0004
Right superiortemporal	48.9	−6.3	−19.1	254.95	7.060	0.0008
Limbic system						
Right insula	32.8	−25.4	19.6	2205.42	16.418	0.0004
Right parahippocampal	33.8	−42.5	−8.3	282.58	9.137	0.0004
Cortical volume						
Frontal lobe						
Right medialorbitofrontal	10.1	34.6	−20	132.91	8.924	0.0004
Parietal lobe						
Right postcentral	57.3	−14.8	38.6	501.32	9.009	0.0004
Temporal lobe						
Left superiortemporal	−49.3	−2.7	−13.7	243.76	8.809	0.0004
Right superiortemporal	50.7	2.1	−14.1	145.36	6.954	0.0004
Right transversetemporal	55.1	−14.2	1.9	102.73	6.310	0.0004

*Note:* Listing of cluster information for regions of brain atrophy related to lower ALPS index. *p*‐value was adjusted for total intracranial volume. The cluster with low size (< 200 mm^2^) has been excluded.

Abbreviations: ALPS, diffusion along the perivascular space; MNI, Montreal Neurological Institute.

### 3.4 Correlation of the ALPS Index With Regional Network Connectivity

**FIGURE 3:**
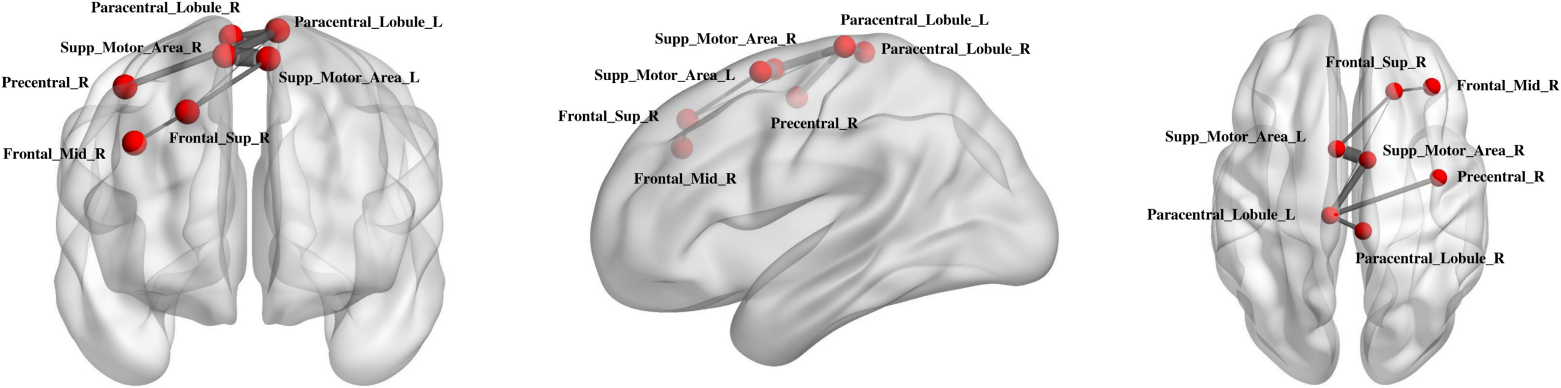
Sub‐networks connectivity related to ALPS index. The connectivity strength of all the presented edges is significantly related to the ALPS index, after correction for multiple testing using the Bonferroni procedure at a false discovery rate of 0.05. The significant nodes and edges were derived from the Network‐Based Statistics analysis performed in GraphVar 2.03a and were visualized using BrainNet Viewer. ALPS, diffusion along the perivascular space.

We further investigated how white matter damage is related to brain atrophy. Figure 3 depicts graphically the sub‐networks constructed with the identified connected nodes and significant edges. In the analysis of whole‐brain network connectivity (Table S6), a 7‐node network was detected in the bilateral parietal lobe and right frontal lobe (Bonferroni‐corrected *p* < 0.05). The lower ALPS index was related to sub‐network dysconnectivity in the midline and right frontal lobe. Considering that a large number of association fibers are distributed in the frontal and parietal lobes' subcortical white matter, the lower ALPS index might be primarily correlated with white matter damage. Moreover, brain atrophy in the corresponding projection areas of association fibers may be the consequence of white matter damage.

## 4 Discussion

This community‐based longitudinal study revealed that a lower ALPS index at baseline was associated with drastic progression of the disruption of white matter microstructural integrity, with the primary location in the subcortical regions where association fibers are distributed. We also found that brain network disconnection related to the lower ALPS index was predominantly located in the right frontal and bilateral parietal lobes, where association fibers are widely distributed [21]. Although the lower ALPS index was remarkably correlated with cortical atrophy in the projection areas of association and projection fibers in cross‐sectional analysis, this correlation was nonsignificant in longitudinal analysis.

Our data suggests a critical association of the ALPS index with the microstructural damage of white matter; furthermore, the ALPS index at baseline showed potential for predicting the progression of white matter microstructural integrity disruption. This aligns with and extends the results of previous studies [22]. On one hand, we assumed that the disruption of cerebral white matter microstructure might be because of diminished glymphatic circulation. Glymphatic dysfunction possibly causes the impedance of perivascular glymphatic fluid and impairs interstitial fluid drainage, which further leads to toxic accumulation and inflammatory response toward myelin [23, 24, 25]. On the other hand, the ALPS index might be influenced not only by glymphatic flow but also by the orientation and microstructural asymmetry of white matter tensor in periventricular regions. Schilling et al. demonstrated that ALPS‐related regions frequently contain complex white matter configurations, including crossing fibers, axonal dispersion, and undulations, which can substantially influence radial diffusivity asymmetry and thereby affect the ALPS index. In particular, the presence of two or more fiber populations within ALPS ROIs challenges the single dominant fiber‐direction assumption and may introduce measurement bias. Therefore, reduced ALPS index values may not exclusively indicate impaired glymphatic or perivascular function, but may also reflect alterations in local white matter microstructural geometry [26]. Recent postmortem evidence further suggests that the ALPS index may be affected by white matter microstructural asymmetry. Using 7.0‐T postmortem DTI in brains with or without Alzheimer's disease pathology, Li et al. quantified the Dxproj/Dyproj ratio and the Dxassoc/Dzassoc ratio in the absence of active perivascular or glymphatic flow, and interpreted these ratios as indices of microstructural asymmetry. They found that these asymmetry measures were still present in postmortem tissue, with projection‐fiber asymmetry associated with amyloid β‐protein (Aβ) pathological status and association‐fiber asymmetry associated with age. After applying a correction based on these postmortem findings to in vivo Alzheimer's Disease Neuroimaging Initiative (ADNI) data, the corrected ALPS index was no longer significantly associated with Aβ Positron Emission Tomography (PET) burden, and its correlation with cognitive performance was attenuated [8]. Therefore, reduced ALPS index values may not exclusively indicate impaired glymphatic or perivascular function, but may also reflect alterations in local white matter microstructural geometry. Together with these findings, our results support the interpretation that the ALPS index is a composite imaging marker influenced by both perivascular fluid dynamics and white matter microstructural integrity, rather than a direct or specific measure of glymphatic function. Therefore, ALPS‐related findings should be interpreted cautiously, particularly in aging and neurodegenerative populations where white matter architecture is frequently altered. Moreover, the brain structural connectome analysis in our study provided converging evidence for this interpretation. Brain regions showing reduced connectivity strength associated with lower ALPS index were mainly located in the right frontal and bilateral parietal areas, which are anatomically interconnected by major association fiber pathways, including the superior longitudinal fasciculus, arcuate fasciculus, and frontoparietal association fiber systems. The substantial spatial overlap between regions of white matter microstructural damage and areas showing reduced structural network connectivity further supports our finding that the ALPS index is significantly associated with white matter microstructural integrity.

In contrast to associations with the white matter, the spatial pattern of cortical atrophy revealed reduced cortical thickness and volume in regions with projections from disrupted white matter tracts rather than remote cortical regions associated with the impairment of glymphatic clearance. This aligns with previous investigations demonstrating a positive correlation between the ALPS index and volumes in the middle frontal regions, amygdala, cingulate, and thalamus, where association and projection fibers are distributed, in cognitively unimpaired older adults [27]. Furthermore, there were no significant findings in longitudinal analysis, consistent with previous reports [22]. Several factors could underlie this lack of association. First, the 5‐year follow‐up duration might be too short for observing significant changes in cortical thickness and volume, suggesting the need for a longer follow‐up. Moreover, the community‐based study population was relatively healthy. The overall severity of cortical atrophy was mild, which may clarify the reason for the observed contrast with the findings of the cortical volume correlation in diseased patients [28, 29]. Future investigation should enroll participants at different stages of various neurodegenerative and vascular diseases. Collectively, our cross‐sectional and longitudinal study indicates the suitability of the ALPS index as an appropriate composite marker of perivascular dynamics and microstructural integrity.

The present study has several strengths that distinguish it from previous studies. First, it was based on a longitudinal community‐based cohort with a relatively large sample size and high‐quality clinical and MRI data. Second, we integrated the ALPS index with multimodal MRI analyses, including TBSS, vertex‐wise analysis with cortical morphology, and structural network connectome analysis. Therefore, compared with previous cross‐sectional studies, our study provides more detailed evidence regarding the temporal and spatial patterns of brain parenchymal damage associated with the ALPS index in our community‐based cohort.

There are, however, some limitations of the present study. First, we excluded participants who could not complete the MRI follow‐up protocol; these individuals were older, inclined to be males, had a greater likelihood of exposure to cardiovascular risk factors, had a larger burden of WMH volume, and had more intense brain atrophy. This aspect may have introduced selection bias. Second, it is noteworthy that the ALPS index only reflects glymphatic activity at the level of the lateral ventricular body. Recent studies using intrathecal CSF tracers confirmed sparse or undetectable exchange between CSF and interstitial fluid in deep white matter regions sampled for the ALPS index [30, 31]. Future studies should validate whether the lower ALPS index in the lateral paraventricular area is a specific symptom of whole brain glymphatic dysfunction. Moreover, Haller et al. also highlighted additional technical limitations, particularly the assumption of a single dominant fiber direction in the regions of complex architecture [32]. Multicompartment models from multi b‐value diffusion MRI are a promising approach to distinguish white matter diffusion properties from perivascular glymphatic activity [33, 34]. These considerations underscore the need for caution when interpreting ALPS values, particularly when used as a standalone marker. Furthermore, our results indicate that the ALPS index alone may not be sufficient to elucidate the dysfunction of the glymphatic circulation pathways (inflow and outflow pathway). Therefore, its greatest utility might be a complementary measure alongside other imaging biomarkers. Future studies are warranted to comprehensively assess the function of the glymphatic circulation using the quantitative volume fraction of perivascular spaces (PVS‐VF), the coupling strength between blood oxygen signals in gray matter and cerebrospinal fluid flow (BOLD‐CSF), periventricular diffusivity (PVeD), the volume of choroid plexus (ChP), free water in white matter (FW‐WM), and meningeal lymphatic vessels (MLVs) [35, 36, 37, 38].

In conclusion, the lower baseline ALPS index is related to drastic progression of disruption of white matter microstructural integrity in the longitudinal community‐based population. The ALPS index could be considered a composite marker of perivascular dynamics and microstructural integrity, rather than a direct indicator of glymphatic function. Considering the present evidence and very few studies on this topic, further longitudinal studies with participants at different stages of various neurodegenerative and vascular diseases are required for comprehensively assessing the relationship of glymphatic function with the ALPS index.

## Author Contributions

Chengqian Li: writing – original draft, investigation, formal analysis. Wen‐Xin Li: data curation, investigation, formal analysis, writing – review and editing. Zi‐Yue Liu: conceptualization, methodology, writing – review and editing. Fei‐Fei Zhai: data curation, investigation, writing – review and editing. Fei Han: conceptualization, investigation, writing – review and editing. Ming‐Li Li: visualization, investigation, writing – review and editing. Li‐Xin Zhou: visualization, investigation, writing – review and editing. Jun Ni: investigation, visualization, writing – review and editing. Ming Yao: visualization, investigation, writing – review and editing. Shu‐Yang Zhang: visualization, investigation, writing – review and editing. Li‐Ying Cui: visualization, investigation, writing – review and editing. Zheng‐Yu Jin: visualization, investigation, writing – review and editing. Yi‐Cheng Zhu: conceptualization, writing – review and editing, validation, funding acquisition, project administration.

## Funding

This study was financially aided by the Noncommunicable Chronic Diseases‐National Science and Technology Major Project (grant numbers: 2023ZD0504900 and 2023ZD0504901), the National High Level Hospital Clinical Research Funding (grant number: 2025‐PUMCH‐C‐012), and the National Natural Science Foundation of China (grant numbers: 82371347 and 82401557).

## Ethics Statement

The Ethics Committee of Peking Union Medical College Hospital provided approval for the study (B‐160 and JS‐2822). A written informed consent form was signed by all participants.

## Consent

The authors have nothing to report.

## Conflicts of Interest

The authors declare no conflicts of interest.

## Supporting information


**Table S1:** Comparison of baseline characteristics between follow‐up participants and lost participants.
**Table S2:** Baseline and longitudinal associations between ALPS index and white matter microstructural measurements.
**Table S3:** Baseline and longitudinal associations between ALPS index and brain structural measurements.
**Table S4:** Listing of white matter fiber tracts damage correlated to lower ALPS index.
**Table S5:** Distribution of progression of brain atrophy with correlation to lower baseline ALPS index.
**Table S6:** Connecting strength identified with significant correlation with ALPS index.
**Figure S1:** Study flow diagram.
**Figure S2:** Distribution of white matter microstructural damage correlated to ALPS index.
**Figure S3:** Distribution of cortical volume regions with significant correlation to ALPS index.

## Data Availability

The data supporting study results can be requested from the corresponding author.

## References

[1] J. M. Klostranec , D. Vucevic , K. D. Bhatia , et al., “Current Concepts in Intracranial Interstitial Fluid Transport and the Glymphatic System: Part I‐Anatomy and Physiology,” Radiology 301, no. 3 (2021): 502–514, 10.1148/radiol.2021202043. 34665028

[2] J. M. Klostranec , D. Vucevic , K. D. Bhatia , et al., “Current Concepts in Intracranial Interstitial Fluid Transport and the Glymphatic System: Part II‐Imaging Techniques and Clinical Applications,” Radiology 301, no. 3 (2021): 516–532, 10.1148/radiol.2021204088. 34698564

[3] P. M. Schirge , R. Perneczky , T. Taoka , et al., “Perivascular Space and White Matter Hyperintensities in Alzheimer's Disease: Associations With Disease Progression and Cognitive Function,” Alzheimer's Research & Therapy 17, no. 1 (2025): 62, 10.1186/s13195-025-01707-9. PMC11917016 40098158

[4] L. Sacchi , F. D'Agata , C. Campisi , et al., “A “Glympse” Into Neurodegeneration: Diffusion MRI and Cerebrospinal Fluid Aquaporin‐4 for the Assessment of Glymphatic System in Alzheimer's Disease and Other Dementias,” Human Brain Mapping 45, no. 12 (2024): e26805, 10.1002/hbm.26805. 39185685 PMC11345637

[5] S. Rudilosso , E. Muñoz‐Moreno , C. Laredo , et al., “Perivascular and Parenchymal Brain Fluid Diffusivity in Patients With a Recent Small Subcortical Infarct,” Neuroradiology 67, no. 3 (2025): 599–611, 10.1007/s00234-025-03546-9. 39853343

[6] J. J. Iliff , M. Wang , Y. Liao , et al., “A Paravascular Pathway Facilitates CSF Flow Through the Brain Parenchyma and the Clearance of Interstitial Solutes, Including Amyloid β,” Science Translational Medicine 4, no. 147 (2012): 147ra111, 10.1126/scitranslmed.3003748. PMC3551275 22896675

[7] T. Taoka , Y. Masutani , H. Kawai , et al., “Evaluation of Glymphatic System Activity With the Diffusion MR Technique: Diffusion Tensor Image Analysis Along the Perivascular Space (DTI‐ALPS) in Alzheimer's Disease Cases,” Japanese Journal of Radiology 35, no. 4 (2017): 172–178, 10.1007/s11604-017-0617-z. 28197821

[8] S. Li , R. Chen , Z. Cao , et al., “Microstructural Bias in the Assessment of Periventricular Flow as Revealed in Postmortem Brains,” Radiology 316, no. 3 (2025): e250753, 10.1148/radiol.250753. 40956166

[9] À. Rovira and D. Pareto , “Diffusion Tensor Imaging Analysis Along the Perivascular Space and Neurodegeneration,” Radiology 316, no. 3 (2025): e252325, 10.1148/radiol.252325. 40956164

[10] S. B. Hladky and M. A. Barrand , “The Glymphatic Hypothesis: The Theory and the Evidence,” Fluids and Barriers of the CNS 19, no. 1 (2022): 9, 10.1186/s12987-021-00282-z. 35115036 PMC8815211

[11] F. Han , L. X. Zhou , J. Ni , et al., “Design of the Shunyi Study on Cardiovascular Disease and Age‐Related Brain Changes: A Community‐Based, Prospective, Cohort Study,” Annals of Translational Medicine 8, no. 23 (2020): 1579, 10.21037/atm-20-4195. 33437778 PMC7791225

[12] F. Alfaro‐Almagro , M. Jenkinson , N. K. Bangerter , et al., “Image Processing and Quality Control for the First 10,000 Brain Imaging Datasets From UK Biobank,” NeuroImage 166 (2018): 400–424, 10.1016/j.neuroimage.2017.10.034. 29079522 PMC5770339

[13] W. X. Li , Z. Y. Liu , F. F. Zhai , et al., “Automated Diffusion‐Weighted Image Analysis Along the Perivascular Space Index Reveals Glymphatic Dysfunction in Association With Brain Parenchymal Lesions,” Human Brain Mapping 45, no. 11 (2024): e26790, 10.1002/hbm.26790. 39037119 PMC11261591

[14] M. Jenkinson , C. F. Beckmann , T. E. Behrens , M. W. Woolrich , and S. M. Smith , “FSL,” NeuroImage 62, no. 2 (2012): 782–790, 10.1016/j.neuroimage.2011.09.015. 21979382

[15] L. Griffanti , G. Zamboni , A. Khan , et al., “BIANCA (Brain Intensity AbNormality Classification Algorithm): A New Tool for Automated Segmentation of White Matter Hyperintensities,” NeuroImage 141 (2016): 191–205, 10.1016/j.neuroimage.2016.07.018. 27402600 PMC5035138

[16] J. D. Kruschwitz , D. List , L. Waller , M. Rubinov , and H. Walter , “GraphVar: A User‐Friendly Toolbox for Comprehensive Graph Analyses of Functional Brain Connectivity,” Journal of Neuroscience Methods 245 (2015): 107–115, 10.1016/j.jneumeth.2015.02.021. 25725332

[17] L. Waller , A. Brovkin , L. Dorfschmidt , D. Bzdok , H. Walter , and J. D. Kruschwitz , “GraphVar 2.0: A User‐Friendly Toolbox for Machine Learning on Functional Connectivity Measures,” Journal of Neuroscience Methods 308 (2018): 21–33, 10.1016/j.jneumeth.2018.07.001. 30026069

[18] Z. Y. Liu , F. F. Zhai , F. Han , et al., “Regional Disruption of White Matter Integrity and Network Connectivity Are Related to Cognition,” Journal of Alzheimer's Disease 89, no. 2 (2022): 593–603, 10.3233/jad-220191. 35912739

[19] M. Xia , J. Wang , and Y. He , “BrainNet Viewer: A Network Visualization Tool for Human Brain Connectomics,” PLoS One 8, no. 7 (2013): e68910, 10.1371/journal.pone.0068910. 23861951 PMC3701683

[20] S. M. Smith , M. Jenkinson , H. Johansen‐Berg , et al., “Tract‐Based Spatial Statistics: Voxelwise Analysis of Multi‐Subject Diffusion Data,” NeuroImage 31, no. 4 (2006): 1487–1505, 10.1016/j.neuroimage.2006.02.024. 16624579

[21] N. Agarwal , L. D. Lewis , L. Hirschler , et al., “Current Understanding of the Anatomy, Physiology, and Magnetic Resonance Imaging of Neurofluids: Update From the 2022 “ISMRM Imaging Neurofluids Study Group” Workshop in Rome,” Journal of Magnetic Resonance Imaging 59, no. 2 (2024): 431–449, 10.1002/jmri.28759. 37141288 PMC10624651

[22] Q. Chen , D. Ge , X. Xu , et al., “Glymphatic Function Associates With Alzheimer's Disease‐Signature Region Volumes, Plasma Biomarkers and White Matter Hyperintensity Progression in Cognitively Unimpaired Older Adults,” Age and Ageing 54, no. 6 (2025): afaf141, 10.1093/ageing/afaf141. 40459343 PMC12131235

[23] X. Zhang , X. Pei , Y. Shi , et al., “Unveiling Connections Between Venous Disruption and Cerebral Small Vessel Disease Using Diffusion Tensor Image Analysis Along Perivascular Space (DTI‐ALPS): A 7‐T MRI Study,” International Journal of Stroke 20, no. 4 (2025): 497–506, 10.1177/17474930241293966. 39402900

[24] B. Zeng , P. Zeng , Y. Xiang , et al., “Expansion of White Matter Hyperintensities Is Associated With the Glymphatic System Dysfunction in Cerebral Small Vessel Disease,” Brain Research Bulletin 227 (2025): 111391, 10.1016/j.brainresbull.2025.111391. 40412489

[25] M. Liao , X. Long , M. Wang , et al., “Disruptions of Deep Medullary Veins and MRI Indices of Glymphatic Function in Cerebral Small Vessel Disease,” Journal of Alzheimer's Disease 107, no. 4 (2025): 1780–1792, 10.1177/13872877251372926. 40899979

[26] K. G. Schilling , A. Newton , C. Tax , et al., “White Matter Geometry Confounds Diffusion Tensor Imaging Along Perivascular Space (DTI‐ALPS) Measures,” Human Brain Mapping 46, no. 10 (2025): e70282, 10.1002/hbm.70282. 40622117 PMC12231058

[27] W. C. Hsiao , H. I. Chang , S. W. Hsu , et al., “Association of Cognition and Brain Reserve in Aging and Glymphatic Function Using Diffusion Tensor Image‐Along the Perivascular Space (DTI‐ALPS),” Neuroscience 524 (2023): 11–20, 10.1016/j.neuroscience.2023.04.004. 37030632

[28] X. Xu , X. Yang , J. Zhang , et al., “Choroid Plexus Free‐Water Correlates With Glymphatic Function in Alzheimer's Disease,” Alzheimer's & Dementia 21, no. 5 (2025): e70239, 10.1002/alz.70239. PMC12092370 40394891

[29] H. Li , M. A. Jacob , M. Cai , et al., “Perivascular Spaces, Diffusivity Along Perivascular Spaces, and Free Water in Cerebral Small Vessel Disease,” Neurology 102, no. 9 (2024): e209306, 10.1212/wnl.0000000000209306. 38626373

[30] G. Ringstad , L. M. Valnes , A. M. Dale , et al., “Brain‐Wide Glymphatic Enhancement and Clearance in Humans Assessed With MRI,” JCI Insight 3, no. 13 (2018): e121537, 10.1172/jci.insight.121537. 29997300 PMC6124518

[31] G. Ringstad , “Glymphatic Imaging: A Critical Look at the DTI‐ALPS Index,” Neuroradiology 66, no. 2 (2024): 157–160, 10.1007/s00234-023-03270-2. 38197950

[32] S. Haller , L. Moy , and Y. Anzai , “Evaluation of Diffusion Tensor Imaging Analysis Along the Perivascular Space as a Marker of the Glymphatic System,” Radiology 310, no. 1 (2024): e232899, 10.1148/radiol.232899. 38289215

[33] Y. Bito , H. Ochi , R. Shirase , W. Yokohama , K. Harada , and K. Kudo , “Low b‐Value Diffusion Tensor Imaging to Analyze the Dynamics of Cerebrospinal Fluid: Resolving Intravoxel Pseudorandom Motion Into Ordered and Disordered Motions,” Magnetic Resonance in Medical Sciences 24, no. 1 (2025): 46–57, 10.2463/mrms.mp.2023-0081. 37899254 PMC11733514

[34] Y. Bito , K. Harada , H. Ochi , and K. Kudo , “Low b‐Value Diffusion Tensor Imaging for Measuring Pseudorandom Flow of Cerebrospinal Fluid,” Magnetic Resonance in Medicine 86, no. 3 (2021): 1369–1382, 10.1002/mrm.28806. 33893650

[35] L. Wu , K. Chen , Z. Zhang , et al., “The Relationship Between Glymphatic Function, White Matter Hyperintensity and Cognition: A Structural Equation Model MRI Study,” CNS Neuroscience & Therapeutics 31, no. 6 (2025): e70478, 10.1111/cns.70478. 40538144 PMC12179404

[36] M. Huang , X. Lyu , P. Wu , and B. Gao , “A Preliminary Study of MRI‐Derived Glymphatic System Indices in Early‐Stage Normal Ageing,” Academic Radiology 32, no. 11 (2025): 6894–6902, 10.1016/j.acra.2025.07.066. 40841256

[37] C. L. Chen , S. J. Son , N. Schweitzer , et al., “Periventricular Diffusivity Reflects APOE ε4‐Modulated Amyloid Accumulation and Cognitive Impairment in the Alzheimer's Disease Continuum,” Alzheimer's & Dementia 21, no. 9 (2025): e70659, 10.1002/alz.70659. PMC12444947 40965291

[38] X. Guo , G. Zhang , Q. Peng , L. Huang , Z. Zhang , and Z. Zhang , “Emerging Roles of Meningeal Lymphatic Vessels in Alzheimer's Disease,” Journal of Alzheimer's Disease 94, no. s1 (2023): S355–s366, 10.3233/jad-221016. PMC10473149 36683509

