# Supplementary material for: Longitudinal Patterns of Brain Parenchymal Damage Associated With the ALPS Index in a Community‐Based Cohort
Source: CNS Neurosci Ther. 2026 Aug 25;32(8):e71107. doi: 10.1002/cns.71107 (PMC13504370; doi:10.1002/cns.71107)
Supplement: Supplementary file 1 [file CNS-32-e71107-s001.docx]

**Supplementary Materials**

**Supplemental Methods**

***MRI protocol***

MRI was performed with a single 3-T Skyra scanner (Siemens, Erlangen, Germany). Three-dimensional time-of-flight magnetic resonance angiography (TOF-MRA) was performed in axial planes with the following parameters: repetition time (TR) = 21 ms, echo time (TE) =3.43 ms, field of view (FOV)= 208 × 229 mm^2^, voxel size = 0.3 × 0.3 × 0.6 mm^3^, and flip angle=18^°^, with a total of 136 axial slices. 3D T1-weighted images using magnetization-prepared rapid gradient-echo sequence (TR = 2530 ms, TE = 3.43 ms, voxel size = 1 × 1 × 1.3 mm^3^, flip angle = 8^°^, 144 sagittal slices), T2- weighted images (TR = 6000 ms, TE = 125 ms, slice thickness = 5 mm, gap = 1 mm, 20 axial slices), fluid-attenuated inversion recovery images (TR = 8500 ms, TE = 81 ms, slice thickness = 5 mm, gap = 1 mm, 20 axial slices) and susceptibility-weighted images (SWI, TR = 20 ms, TE = 27 ms, slice thickness =1.5 mm, flip angle =15^°^, 80 axial slices) were also acquired.

Diffusion tensor images (DTI) were acquired using a single-shot spin echo-planar imaging sequence covering the whole brain with the following parameters: 62 axial slices, slice thickness = 2.2 mm without gap; repetition time (TR) = 8,000 ms; echo time (TE) = 89 ms; flip angle = 90^°^; 30 diffusion directions with b = 0 and 1000 s/mm^2^; acquisition matrix = 128 × 128; FOV = 280 × 280 mm^2^, average = 2.

***Assessment of covariates***

Hypertension was defined as self-reported hypertension or treatment with antihypertensive medication or systolic blood pressure ≥140 mmHg or diastolic blood pressure ≥90 mmHg. Diabetes mellitus was defined as self-reported diabetes or the use of oral antidiabetic drugs or insulin or fasting serum glucose ≥7.0 mmol/L, or hemoglobin A1c ≥6.5%. Hyperlipidemia was defined as self-reported hyperlipidemia or treatment with lipid-lowering medication or total cholesterol >5.2 mmol/L or low-density lipoprotein cholesterol >3.62 mmol/L. Smoking history was defined as a current or former smoker. Alcohol consumption history was defined based on participants self-reported alcohol intake.

***Reference***

1. Han F, Zhou LX, Ni J, et al. Design of the Shunyi study on cardiovascular disease and age-related brain changes: a community-based, prospective, cohort study. Ann Transl Med. Dec 2020;8(23):1579. doi:10.21037/atm-20-4195

**Table S1. Comparison of baseline characteristics between follow-up participants and lost participants.**

| Variables | Total (n = 1120) | Follow-up (n=682) | Missing (n=438) | P value ^a^ |
| --- | --- | --- | --- | --- |
| Demographic characteristics |  |  |  |  |
| Age, mean (SD), year | 56.99 (9.26) | 55.02 (7.98) | 60.07 (10.23) | <0.001 |
| Male, No. (%) | 394 (35.18) | 223 (32.70) | 171 (39.04) | 0.035 |
| Hypertension, No. (%) | 580 (51.79) | 326 (47.80) | 254 (57.99) | 0.001 |
| Diabetes mellitus, No. (%) | 180 (16.07) | 86 (12.61) | 94 (21.46) | <0.001 |
| Hyperlipidemia, No. (%) | 536 (47.86) | 318 (46.63) | 218 (49.77) | 0.334 |
| Smoking history, No. (%) | 237 (21.16) | 124 (18.18) | 113 (25.80) | 0.003 |
| Alcohol consumption history, No. (%) | 247 (22.05) | 148 (21.70) | 99 (22.60) | 0.778 |
| Baseline neuroimaging characteristics |  |  |  |  |
| ALPS index | 1.41 (0.13) | 1.42 (0.12) | 1.38 (0.14) | <0.001 |
| BPF, mean (SD), % | 75.26 (2.97) | 75.69 (2.59) | 74.60 (3.38) | <0.001 |
| GMF, mean (SD), % | 34.40 (1.64) | 34.60 (1.53) | 34.08 (1.75) | <0.001 |
| WMF, mean (SD), % | 32.16 (1.91) | 32.36 (1.77) | 31.87 (2.07) | <0.001 |
| Hippocampus fraction, mean (SD), % | 0.57 (0.05) | 0.57 (0.05) | 0.56 (0.05) | 0.002 |
| WMH volume, median (interquartile range), mL | 8.00 (7.56, 8.60) | 7.89 (7.50, 8.38) | 8.25 (7.69, 8.93) | <0.001 |
| Fractional anisotropy, mean (SD) | 0.40 (0.01) | 0.398 (0.008) | 0.396 (0.009) | <0.001 |
| Mean diffusivity, mean (SD), ×10^−3^ mm^2^/s | 0.79 (0.02) | 0.793 (0.021) | 0.798 (0.023) | 0.001 |
| Axial diffusivity, mean (SD), ×10^−3^ mm^2^/s | 1.16 (0.03) | 1.157 (0.024) | 1.162 (0.027) | 0.010 |
| Radial diffusivity, mean (SD), ×10^−3^ mm^2^/s | 0.61 (0.02) | 0.610 (0.020) | 0.616 (0.022) | <0.001 |

**Abbreviations:** ALPS, diffusion along the perivascular space; BPF, brain parenchymal fraction, the sum of gray matter and white matter volumes/total intracranial volume; GMF, gray matter fraction, gray matter volume/total intracranial volume; WMF, white matter fraction, white matter volume/total intracranial volume; Hippocampus fraction, hippocampus volume/total intracranial volume; WMH, white matter hyperintensity; SD, standard deviation.

^a^ Significance test between the follow-up group and lost to follow-up group.

**Table S2. Baseline and longitudinal associations between** **ALPS index and white matter microstructural measurements.**

| Variables | Model 1 | | | Model 2 | | | Model 3 | | |
| --- | --- | --- | --- | --- | --- | --- | --- | --- | --- |
|  | β (SE) /  RR (95%CI) | p | p-FDR | β (SE) /  RR (95%CI) | p | p-FDR | β (SE) /  RR (95%CI) | p | p-FDR |
| **Baseline** |  |  |  |  |  |  |  |  |  |
| FA | 0.014 (0.002) | **<0.001** | **<0.001** | 0.008 (0.002) | **<0.001** | **<0.001** | 0.008 (0.002) | **<0.001** | **<0.001** |
| MD | -0.001 (0.001) | **<0.001** | **<0.001** | -0.001 (0.001) | **<0.001** | **<0.001** | -0.001 (0.001) | **<0.001** | **<0.001** |
| AD | -0.001 (0.001) | **<0.001** | **<0.001** | -0.001 (0.001) | **<0.001** | **<0.001** | -0.001 (0.001) | **<0.001** | **<0.001** |
| RD | -0.001 (0.001) | **<0.001** | **<0.001** | -0.001 (0.001) | **<0.001** | **<0.001** | -0.001 (0.001) | **<0.001** | **<0.001** |
| **Longitudinal** |  |  |  |  |  |  |  |  |  |
| FA |  |  |  |  |  |  |  |  |  |
| ALPS | 1.027 (1.014, 1.041) | **<0.001** | **<0.001** | 1.019 (1.006, 1.033) | **0.004** | **0.004** | 1.020 (1.007, 1.033) | **0.003** | **0.003** |
| ALPS * Time | 0.999 (0.991, 1.006) | 0.697 | 0.697 | 0.999 (0.991, 1.006) | 0.696 | 0.696 | 0.999 (0.991, 1.006) | 0.696 | 0.696 |
| MD |  |  |  |  |  |  |  |  |  |
| ALPS | 0.958 (0.942, 0.974) | **<0.001** | **<0.001** | 0.972 (0.956, 0.989) | **<0.001** | **<0.001** | 0.972 (0.956, 0.988) | **<0.001** | **<0.001** |
| ALPS * Time | 0.994 (0.981, 1.008) | 0.417 | 0.697 | 0.994 (0.981, 1.008) | 0.418 | 0.696 | 0.994 (0.981, 1.008) | 0.418 | 0.696 |
| AD |  |  |  |  |  |  |  |  |  |
| ALPS | 0.968 (0.955, 0.981) | **<0.001** | **<0.001** | 0.978 (0.965, 0.991) | **0.001** | **0.002** | 0.978 (0.965, 0.991) | **<0.001** | **<0.001** |
| ALPS * Time | 0.994 (0.982, 1.005) | 0.275 | 0.697 | 0.994 (0.982, 1.005) | 0.275 | 0.696 | 0.994 (0.982, 1.005) | 0.275 | 0.696 |
| RD |  |  |  |  |  |  |  |  |  |
| ALPS | 0.950 (0.930, 0.970) | **<0.001** | **<0.001** | 0.967 (0.947, 0.987) | **0.002** | **0.003** | 0.967 (0.947, 0.987) | **0.001** | **0.001** |
| ALPS * Time | 0.995 (0.980, 1.011) | 0.562 | 0.697 | 0.995 (0.980, 1.011) | 0.562 | 0.696 | 0.995 (0.980, 1.011) | 0.563 | 0.696 |

Model 1 was univariate. Model 2 was adjusted for age and sex. Model 3 was adjusted for age, sex and vascular risk factors (hypertension, diabetes, hyperlipidemia, smoking and alcohol consumption history). ALPS, diffusion along the perivascular space; FA, fractional anisotropy; MD, mean diffusivity; AD, axial diffusivity; RD, radial diffusivity; SE, standard error; RR, relative ratio; CI, confidence interval; FDR, false discovery rate.

Bold values indicate *P*<0.05 or *P*<0.05 after FDR correction.

| Variables | Model 1 | | | Model 2 | | | Model 3 | | |
| --- | --- | --- | --- | --- | --- | --- | --- | --- | --- |
|  | β (SE)/ Abs. Change (95% CI) | p | p-FDR | β (SE) / Abs. Change (95% CI) | p | p-FDR | β (SE) / Abs. Change (95% CI) | p | p-FDR |
| **Baseline** |  |  |  |  |  |  |  |  |  |
| BPF | 0.081 (0.006) | **<0.001** | **<0.001** | 0.038 (0.006) | **<0.001** | **<0.001** | 0.037 (0.006) | **<0.001** | **<0.001** |
| GMF | 0.032 (0.004) | **<0.001** | **<0.001** | 0.010 (0.004) | **0.006** | **0.006** | 0.010 (0.004) | **0.006** | **0.006** |
| WMF | 0.042 (0.004) | **<0.001** | **<0.001** | 0.026 (0.004) | **<0.001** | **<0.001** | 0.025 (0.004) | **<0.001** | **<0.001** |
| Hippocampus fraction | 0.0009 (0.0001) | **<0.001** | **<0.001** | 0.0004 (0.001) | **<0.001** | **<0.001** | 0.0004 (0.0001) | **<0.001** | **<0.001** |
| WMH fraction | -0.0017 (0.0002) | **<0.001** | **<0.001** | -0.0011 (0.0001) | **<0.001** | **<0.001** | -0.0011 (0.0001) | **<0.001** | **<0.001** |
| **Longitudinal** |  |  |  |  |  |  |  |  |  |
| BPF |  |  |  |  |  |  |  |  |  |
| ALPS | 0.073 (0.043, 0.102) | **<0.001** | **<0.001** | 0.037 (0.008, 0.066) | **0.013** | **0.016** | 0.037 (0.008, 0.066) | **0.012** | **0.015** |
| ALPS * Time | 0.015 (-0.001, 0.031) | 0.060 | 0.075 | 0.015 (-0.001, 0.031) | 0.059 | 0.074 | 0.015 (-0.001, 0.031) | 0.059 | 0.074 |
| GMF |  |  |  |  |  |  |  |  |  |
| ALPS | 0.018 (0.004, 0.032) | **0.012** | **0.012** | 0.003 (-0.011, 0.016) | 0.696 | 0.696 | 0.003 (-0.011, 0.017) | 0.656 | 0.656 |
| ALPS * Time | 0.005 (-0.002, 0.013) | 0.179 | 0.179 | 0.005 (-0.002, 0.013) | 0.179 | 0.179 | 0.005 (-0.002, 0.013) | 0.179 | 0.179 |
| WMF |  |  |  |  |  |  |  |  |  |
| ALPS | 0.038 (0.019, 0.056) | **<0.001** | **<0.001** | 0.026 (0.008, 0.045) | **0.006** | **0.010** | 0.026 (0.007, 0.045) | **0.007** | **0.012** |
| ALPS * Time | 0.010 (0.002, 0.019) | **0.021** | **0.037** | 0.010 (0.002, 0.019) | **0.021** | **0.037** | 0.010 (0.002, 0.019) | **0.021** | **0.037** |
| Hippocampus fraction |  |  |  |  |  |  |  |  |  |
| ALPS | 0.045 (0.031, 0.060) | **<0.001** | **<0.001** | 0.022 (0.009, 0.036) | **0.001** | **0.003** | 0.022 (0.009, 0.036) | **0.001** | **0.003** |
| ALPS * Time | 0.006 (0.001, 0.011) | **0.016** | **0.037** | 0.006 (0.001, 0.011) | **0.016** | **0.037** | 0.006 (0.001, 0.011) | **0.016** | **0.037** |
| WMH fraction |  |  |  |  |  |  |  |  |  |
| ALPS | -0.052(-0.069, -0.034) | **<0.001** | **<0.001** | -0.040 (-0.055, -0.024) | **<0.001** | **<0.001** | -0.040 (-0.055, -0.025) | **<0.001** | **<0.001** |
| ALPS * Time | 0.009 (0.001, 0.016) | **0.022** | **0.037** | 0.009 (0.001, 0.016) | **0.022** | **0.037** | 0.009 (0.001, 0.016) | **0.022** | **0.037** |

**Table S3. Baseline and longitudinal associations between ALPS index and brain structural measurements.**

Model 1 was univariate. Model 2 was adjusted for age and sex. Model 3 was adjusted for age, sex and vascular risk factors (hypertension, diabetes, hyperlipidemia, smoking and alcohol consumption history). The Abs. Change indicated absolute change in proportion, which was calculated by back - transforming logit - scaled estimates to the original proportion scale. ALPS, diffusion along the perivascular space; BPF, brain parenchymal fraction, the sum of gray matter and white matter volumes/total intracranial volume; GMF, gray matter fraction, gray matter volume/total intracranial volume; WMF, white matter fraction, white matter volume/total intracranial volume; Hippocampus fraction, bilateral hippocampus volume/total intracranial volume; WMH, white matter hyperintensity; SE, standard error; CI, confidence interval; FDR, false discovery rate.

Bold values indicate *P*<0.05 or *P*<0.05 after FDR correction.

**Table S4. Listing of white matter fiber tracts damage correlated to lower ALPS index.**

| DTI parameters | White matter fiber tracts  (JHU WM tractography atlas) | Voxel coordinates of local maxima  (MNI coordinates) | | | Cluster size  (Voxels) | t score | P value |
| --- | --- | --- | --- | --- | --- | --- | --- |
|  |  | X | Y | Z |  |  |  |
| **FA** | Body of corpus callosum | 0.0 | -2.0 | 14.0 | 56003 | 13.647 | <0.05 |
|  | Anterior limb of internal capsule R | 23.0 | 7.0 | 16.0 | 482 | 8.351 | <0.05 |
|  | Anterior limb of internal capsule L | -22.0 | 6.0 | 18.0 | 420 | 8.279 | <0.05 |
|  | Middle cerebellar peduncle | -22.0 | -62.0 | -35.0 | 245 | 5.138 | <0.05 |
|  | External capsule R | 30.0 | 16.0 | -1.0 | 152 | 5.817 | <0.05 |
|  | Superior longitudinal fasciculus R | 33.0 | -32.0 | 36.0 | 123 | 6.512 | <0.05 |
|  | Uncinate fasciculus L | -28.0 | -1.0 | -12.0 | 119 | 5.842 | <0.05 |
| **AD** | Splenium of corpus callosum | -38.0 | 2.0 | 21.0 | 17919 | 1.532 | <0.05 |
|  | Fornix (column and body of fornix) | -10.0 | -13.0 | 15.0 | 1299 | 2.387 | <0.05 |
| **RD** | Body of corpus callosum | 15.0 | 11.0 | 53.0 | 75896 | 1.327 | <0.05 |
|  | Middle cerebellar peduncle | 30.0 | -50.0 | -29.0 | 4248 | 2.215 | <0.05 |
|  | Superior cerebellar peduncle R | 14.0 | -36.0 | -39.0 | 313 | 2.371 | <0.05 |
|  | Corticospinal tract L | -1.0 | -23.0 | -36.0 | 135 | 2.658 | <0.05 |

Cluster sizes and locations for voxels with significantly decreased FA and increased AD, RD values correlated to lower ALPS index. P value was adjusted for age and sex. The cluster with low size (voxels <100) has been excluded. DTI, diffusion tensor imaging; ALPS, diffusion along the perivascular space; JHU WM tractography atlas, John Hopkins University white-matter tractography atlas; MNI, Montreal Neurological Institute; FA, fractional anisotropy; AD, axial diffusivity; RD, radial diffusivity; L, left hemisphere; R, the right hemisphere.

**Table S5. Distribution of progression of brain atrophy with correlation to lower baseline ALPS index.**

| Brain regions | MNI coordinates | | | Size (mm^2^) | Effect size | P value |
| --- | --- | --- | --- | --- | --- | --- |
|  | X | Y | Z |  |  |  |
| **Cortical thickness** |  |  |  |  |  |  |
| Frontal lobe |  |  |  |  |  |  |
| left paracentral | -8.5 | -18.9 | 51.7 | 73.82 | -3.650 | 0.611 |
| left rostralmiddlefrontal | -39.5 | 26.6 | 15.6 | 62.94 | 2.616 | 0.666 |
| left rostralmiddlefrontal | -23.1 | 50.8 | 9.8 | 53.38 | 2.437 | 0.723 |
| Parietal lobe |  |  |  |  |  |  |
| right superiorparietal | 14.4 | -80.7 | 38.9 | 310 | 2.715 | 0.179 |
| right postcentral | 30.1 | -26.5 | 59.8 | 77.08 | 2.185 | 0.632 |
| right inferiorparietal | 45.7 | -54.1 | 13.7 | 73.68 | 3.011 | 0.644 |
| Temporal lobe |  |  |  |  |  |  |
| left inferiortemporal | -49.3 | -41.2 | -18.3 | 90.8 | 3.141 | 0.535 |
| **Cortical volume** |  |  |  |  |  |  |
| Frontal lobe |  |  |  |  |  |  |
| right caudalmiddlefrontal | 38.1 | 6.1 | 38 | 110.6 | 2.956 | 0.391 |
| left caudalmiddlefrontal | -39.2 | 11.5 | 39.5 | 65.53 | 2.349 | 0.498 |
| right paracentral | 13.5 | -16.2 | 43.1 | 61.4 | -3.224 | 0.563 |
| Parietal lobe |  |  |  |  |  |  |
| left postcentral | -40.4 | -15.5 | 30 | 197.46 | 4.179 | 0.204 |
| right inferiorparietal | 42.8 | -54.4 | 13.4 | 152.41 | 4.565 | 0.299 |
| right postcentral | 28.6 | -26.5 | 59.7 | 64.88 | 2.375 | 0.546 |
| Temporal lobe |  |  |  |  |  |  |
| left superiortemporal | -36.7 | -33.8 | 11.2 | 65.1 | 2.563 | 0.502 |
| left bankssts | -47.6 | -38.5 | 0.5 | 53.66 | 3.460 | 0.559 |
| right bankssts | 43.7 | -33.3 | 1.9 | 188.12 | 4.833 | 0.250 |
| Limbic system |  |  |  |  |  |  |
| right insula | 33.5 | -28.1 | 15.4 | 108.63 | 2.871 | 0.397 |

Listing of cluster information for regions of progression of brain atrophy related to lower baseline ALPS index. P value was adjusted for total intracranial volume. The cluster with low size (< 50 mm^2^) has been excluded. ALPS, diffusion along the perivascular space; MNI, Montreal Neurological Institute.

**Table S6. Connecting strength identified with significant correlation with ALPS index**

|  | β | t score | P value |
| --- | --- | --- | --- |
| MFG.R-SFGdor.R | 0.1411 | 4.75 | 0.0002 |
| SFGdor.R-SMA.L | 0.1498 | 5.05 | ＜.0001 |
| SFGdor.R-PCL.L | 0.12 | 4.03 | ＜.0001 |
| SMA.L-SMA.R | 0.2649 | 9.15 | ＜.0001 |
| SMA.R-PCL.L | 0.1916 | 6.50 | ＜.0001 |
| PCL.L-PreCG.R | 0.1711 | 5.79 | ＜.0001 |
| PCL.L-PCL.R | 0.1646 | 5.56 | ＜.0001 |

Abbreviations: MFG.R, Middle frontal gyrus (Right); SFGdor.R, Superior frontal gyrus, dorsolateral (Right); SMA.L, Supplementary motor area (Left); SMA.R, Supplementary motor area (Right); PCL.L, Paracentral lobule (Left); PCL.R, Paracentral lobule (Right); PreCG.R, Precentral gyrus (Right); ALPS, diffusion along the perivascular space. The analysis was performed using a permutation-based statistical tool in GraphVar 2.03a. The number of the permutation tests was set at 5000 and all p values were Bonferroni corrected.

**Figure S1. Study Flow Diagram**

Abbreviations: MRI, magnetic resonance imaging; ALPS, diffusion along the perivascular space.

**Figure S2. Distribution of white matter microstructural damage correlated to ALPS index.**

The red-yellow map and blue map show locations on the white matter skeleton where FA decrease, or increase in MD, AD, and RD is correlated with lower ALPS index, respectively (threshold-free cluster enhancement corrected P<0.05, adjusted for age and sex). ALPS, diffusion along the perivascular space; FA, fractional anisotropy; MD, mean diffusivity; AD axial diffusivity; RD, radial diffusivity.

**Figure S3. Distribution of cortical volume regions with significant correlation to ALPS index**

Clusters of cortical volume significantly associated with ALPS index, including right postcentral, right medialorbitofrontal, bilateral superiortemporal, and right transversetemporal (Bonferroni-corrected with Monte Carlo simulation for cluster p-value <0.05 and adjusted for total intracranial volume). The displayed color bar is in the logarithmic scale of p values (−log10 [p value]). ALPS, diffusion along the perivascular space.
